# Neuroprotective properties of queen bee acid by autophagy induction

**DOI:** 10.1007/s10565-021-09625-w

**Published:** 2021-08-27

**Authors:** Guadalupe Martínez-Chacón, Marta Paredes-Barquero, Sokhna M.S Yakhine-Diop, Elisabet Uribe-Carretero, Ariadna Bargiela, María Sabater-Arcis, José Morales-García, Jesús Alarcón-Gil, Eva Alegre-Cortés, Saray Canales-Cortés, Mario Rodríguez-Arribas, Pedro Javier Camello, José Manuel Bravo-San Pedro, Ana Perez-Castillo, Rubén Artero, Rosa A. Gonzalez-Polo, José M. Fuentes, Mireia Niso-Santano

**Affiliations:** 1 Departamento de Bioquímica y Biología Molecular y Genética, Facultad de Enfermería y Terapia Ocupacional, Universidad de Extremadura, Avda de la Universidad s/n, 10003 Cáceres, Spain; 2 Centro de Investigación Biomédica en Red de Enfermedades Neurodegenerativas (CIBERNED), Madrid, Spain; 3 Instituto Universitario de Investigación Biosanitaria de Extremadura (INUBE), Cáceres, Spain; 4 Translational Genomics Group, Incliva Health Research Institute, Valencia, Spain; 5 Interdisciplinary Research Structure for Biotechnology and Biomedicine (ERI BIOTECMED), University of Valencia, Valencia, Spain; 6 CIPF-INCLIVA Joint Unit, Valencia, Spain; 7 Departamento de Biología Celular, Facultad de Medicina, Universidad Complutense de Madrid, Madrid, Spain; 8 Instituto de Investigaciones Biomédicas (CSIC-UAM) “Alberto Sols” (CSIC-UAM), Madrid, Spain; 9 Instituto Ramón y Cajal de Investigación Sanitaria (IRYCIS), Madrid, Spain; 10 Departamento de Fisiología, Facultad de Veterinaria, Universidad de Extremadura, Cáceres, Spain; 11 Instituto Universitario de Biomarcadores de Patologías Metabólicas, Cáceres, Spain; 12 Departamento de Fisiología, Facultad de Medicina, Universidad Complutense de Madrid, Madrid, Spain

**Keywords:** Autophagy, Longevity, Neurodegeneration, Parkinson’s disease, QBA, SIRT1

## Abstract

Autophagy is a conserved intracellular catabolic pathway that removes cytoplasmic components to contribute to neuronal homeostasis. Accumulating evidence has increasingly shown that the induction of autophagy improves neuronal health and extends longevity in several animal models. Therefore, there is a great interest in the identification of effective autophagy enhancers with potential nutraceutical or pharmaceutical properties to ameliorate age-related diseases, such as neurodegenerative disorders, and/or promote longevity. Queen bee acid (QBA, 10-hydroxy-2-decenoic acid) is the major fatty acid component of, and is found exclusively in, royal jelly, which has beneficial properties for human health. It is reported that QBA has antitumor, anti-inflammatory, and antibacterial activities and promotes neurogenesis and neuronal health; however, the mechanism by which QBA exerts these effects has not been fully elucidated. The present study investigated the role of the autophagic process in the protective effect of QBA. We found that QBA is a novel autophagy inducer that triggers autophagy in various neuronal cell lines and mouse and fly models. The beclin-1 (BECN1) and mTOR pathways participate in the regulation of QBA-induced autophagy. Moreover, our results showed that QBA stimulates sirtuin 1 (SIRT1), which promotes autophagy by the deacetylation of critical ATG proteins. Finally, QBA-mediated autophagy promotes neuroprotection in Parkinson’s disease in vitro and in a mouse model and extends the lifespan of *Drosophila melanogaster*. This study provides detailed evidences showing that autophagy induction plays a critical role in the beneficial health effects of QBA.

**Supplementary Information:**

The online version contains supplementary material available at 10.1007/s10565-021-09625-w.

## Introduction

Macroautophagy (hereafter referred to as autophagy) is an evolutionarily conserved degradative pathway that is rapidly upregulated to maintain cellular and organismal homeostasis when cells are exposed to stressful conditions (Mizushima and Komatsu 2011). Autophagy is characterized by the encompassing of cytoplasmic structures in a double-membrane vesicle called the autophagosome that fuses with lysosomes where their content is degraded by acidic hydrolases. This dynamic process is highly regulated by specific autophagy-related (ATG) genes (Feng et al. 2014). Moreover, various signaling pathways contribute to the outcome of autophagy regulation. Mammalian target of rapamycin (mTOR) kinase is the major signal inhibitor that prevents autophagy in the presence of growth factor and nutrient availability (Liu and Sabatini 2020). Interestingly, sirtuin 1 (SIRT1) is another nutrient sensor that can regulate autophagy through the deacetylation of ATG proteins such as ATG5, ATG6, ATG7, and ATG8/light-chain microtubule-associated protein (LC3) (Lee et al. 2008). In mammals, beclin-1 (BECN1) is a key component of class III phosphatidylinositol 3 kinase complex (PI3K-III), which plays a critical role in autophagy through the generation of phosphatidylinositol 3 phosphate (PI3P) for the nucleation of the phagophore and autophagosome biogenesis (Axe et al. 2008).

Most cells in the brain are postmitotic and require well-regulated protein quality control systems to avoid the accumulation of altered proteins and organelles (Chu 2019). In fact, a common pathological hallmark of neurodegenerative disorders is the accumulation of abnormal or misfolded proteins (Morimoto and Cuervo 2014). Increasing evidence shows that dysfunctional autophagy is associated with several neuronal disorders (Hara et al. 2006; Komatsu et al. 2006). Therefore, optimal activation of autophagy may be an essential strategy to either prevent or degrade the protein aggregation and may be promising in the treatment or prevention of neurodegeneration (Menzies et al. 2017). In subsequent years, considerable interest has emerged in the identification of autophagy-regulating compounds as potential therapeutic agents in neurodegenerative diseases. In mammals, many studies have reported close relationships between nutritional factors and autophagy regulation (Castagnaro et al. 2016; Niso-Santano et al. 2015; Sheng et al. 2017). Caloric restriction (CR) is a strong physiological inducer of autophagy (Mercken et al. 2013). The signaling pathways regulating CR-mediated autophagy have been extensively studied. These pathways include the inhibition of mTOR activity and the activation of AMP-activated protein kinase (AMPK) and SIRT1 (Cohen et al. 2004; Efeyan et al. 2015; Kim et al. 2011). Furthermore, BECN1 is essential for activating the pro-autophagic PI3K-III complex in response to nutrient starvation (McKnight and Zhenyu 2013). In fact, CR and several natural compounds that mimic the biochemical effect of CR have been shown to promote beneficial effects and longevity (Madeo et al. 2019).

Natural bee products including honey, propolis, and royal jelly (RJ) have received increasing interest due to their therapeutic properties (Pasupuleti et al. 2017). RJ and its derived compounds possess biological activities that improve health and enhance longevity in several animal models (Honda et al. 2011; Inoue et al. 2003; Kunugi and Mohammed Ali 2019; Qiu et al. 2020; Wan et al. 2018; Xin et al. 2016). It has been reported that the lipid fraction of RJ, including 10-hydroxy-2-decenoic acid (10-H_2_DA, namely, queen bee acid (QBA)) and 10-hydroxydecanoic acid (10-HDA), may be responsible for its bioactive properties (Yang et al. 2010). QBA is the exclusive fatty acid of RJ, representing 40% of total fatty acid composition, and it is used as a quality marker of RJ (Li et al. 2013). Several studies have reported that QBA has anti-inflammatory, anticancer, and anti-angiogenic activities (Izuta et al. 2009; Peng et al. 2017; Sugiyama et al. 2012; Yang et al. 2018; You et al. 2019a). There are no strong evidences indicating that peripheral QBA crosses the blood-brain barrier (BBB); however, it has been shown that its administration to animal models promotes neurogenesis and neuronal health (Hattori et al. 2007). Although several studies have investigated the biological effects of QBA, its mechanism of action has not been fully elucidated. To address this issue, the present study investigated whether QBA exerts its healthy effects through autophagy induction by activating the molecular mechanisms of CR.

## Results

### Discovery of QBA as an autophagy modulator

**Fig. 1:**
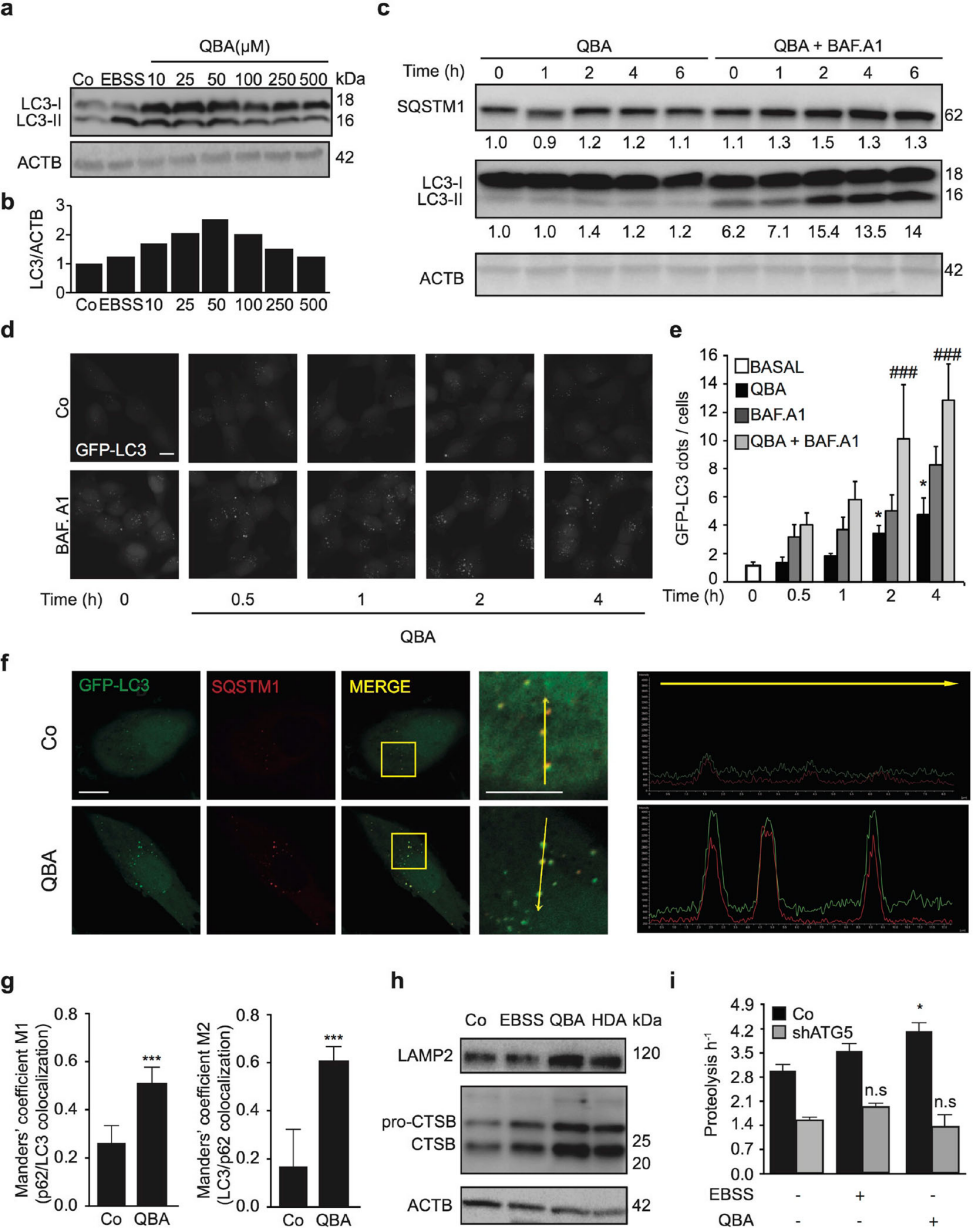
Induction of autophagy by QBA. (a–b) H4 cells were cultured in control conditions (Co), incubated with nutrient-free (EBSS) medium, or treated with QBA (10, 25, 50, 100, 250, and 500 μM) for 4 h followed by the assessment of LC3 lipidation by Western blot. ACTB was used as a loading control, and densitometry was employed to quantify the abundance of lipidated LC3 (LC3-II). (c) SH-SY5Y and (d) H4 cells stably expressing GFP-LC3 were maintained in control conditions, treated with QBA (50 μM) alone or in combination with 100 nM Bafilomycin A1 (BAF.A1) at the indicated times. (c) LC3 lipidation and SQSTM1 degradation were assessed by immunoblotting. ACTB levels were used as a loading control. Densitometry was employed to quantify the abundance of LC3-II and SQSTM1. (e) The number of cytoplasmic GFP-LC3^+^ dots per cell was quantified by fluorescence microscopy in H4-GFP-LC3 cells. Scale bar = 10μm. Data are the means ± SD of at least three independent experiments (*p <0.05 versus untreated cells) ^###^p <0.001 versus BAF.A1. conditions. (f–g) H4-GFP-LC3 cells were maintained in control conditions (Co) or 50 μM QBA for 4 h. Thereafter, p62-LC3 colocalization was detected by immunofluorescence. (f) Representative confocal images of GFP-LC3 (green) and p62 (red) and plots of fluorescence represent the distribution of fluorescence intensities along the line profile (yellow) Arrows indicate localized or unlocalized LC3 to p62. Scale bar = 10 μm. (g) The graphs represent the average of the Mander´s coefficient for the colocalization of p62 to LC3 (M1) or LC3 to p62 (M2) plus standard deviation. Symbols indicate significance at ***p <0.001versus untreated cells. (h) SH-5YSY cells were cultured in control conditions (Co), EBSS medium, or treated with 50 μM QBA or 10HDA for 4 h followed by the assessment of LAMP2 and Cathepsin B levels by immunoblotting. ACTB was used as a loading control. (i) Control (Co) and shATG5 SH-SY5Y cells were cultured in control conditions (Co), incubated with EBSS medium or treated with 50 μM QBA for 4 h. Long-lived protein degradation was determined by pulse chase assay using radioactive valine as indicated in the “Materials and methods” section. Columns indicate means ± SD. Symbols indicate significant, *p <0.05 and non-significant (n.s.) at p >0.05 versus untreated cells.

To determine whether QBA induces autophagy, we exposed H4 (Fig. 1a, b) and glial U251 (Fig. S1a) cells to several concentrations of this fatty acid followed by measurement of lipidated LC3 (LC3-II) levels by Western blotting. We found that QBA treatment enhanced the amount of LC3-II compared to control cells in both cell lines. Next, we monitored autophagy flux in H4 cells that stably express a green fluorescent protein (GFP)-LC3 chimera treated with QBA in the presence or absence of Bafilomycin A1 (BAF.A1, a lysosomal inhibitor) at different time points. We observed that QBA increased LC3 puncta compared to basal conditions, which were further significantly enhanced in the presence of BAF.A1 at 2 and 4 h of treatment (Fig. 1d, e). These results were confirmed in SH-SY5Y cells by Western blotting using BAF.A1 (Fig. 1c and fig. S1b). Similar to QBA, 10-hydroxydecanoic acid (HDA) also increased autophagy flux in H4-GFP-LC3 cells (Fig. S1c, d). In addition, QBA increased the colocalization of p62 with LC3-positive vacuoles (Fig. 1f, g) in GFP-LC3 H4 cells. This autophagy activation induced by QBA was accompanied by an increase in the number of lysosomes. As shown in Fig. 1h, QBA treatment promoted the accumulation of lysosomal-associated membrane protein-2 (LAMP2) and the Cathepsin B mature isoform suggesting enhanced lysosomal biogenesis. To confirm the effect of QBA on the induction of autophagy, the catabolism of long-lived proteins was determined by a pulse chase assay. As shown in Fig. 1i, QBA treatment remarkably enhanced the turnover of labeled long-lived proteins. Moreover, the depletion of ATG5 in SH-SY5Y cells exhibited a decreased long-lived protein degradation (Fig. 1i) and a diminution of LC3-II levels in both *atg5*-deficient SH-SY5Y/MEF cells (Fig S1e, f). Similar results were observed in HDA-treated cells (Fig. S1e). These results clearly indicated that QBA induces autophagy in vitro in several cell lines, including neurons.

### QBA-mediated autophagy is BECN1/mTOR-dependent

To understand the molecular pathways implicated in QBA-induced autophagy, we studied the involvement of BECN1 protein and the mTOR pathway. BECN1 interacts with several ATG proteins to generate the PI3K-III complex that is required for autophagosome biogenesis (Axe et al. 2008). We studied the implications of the PI3K-III complex in the pro-autophagic activity of QBA by reducing BECN1 expression through gene silencing assays and monitoring changes in LC3 lipidation by Western blotting.

**Fig. 2:**
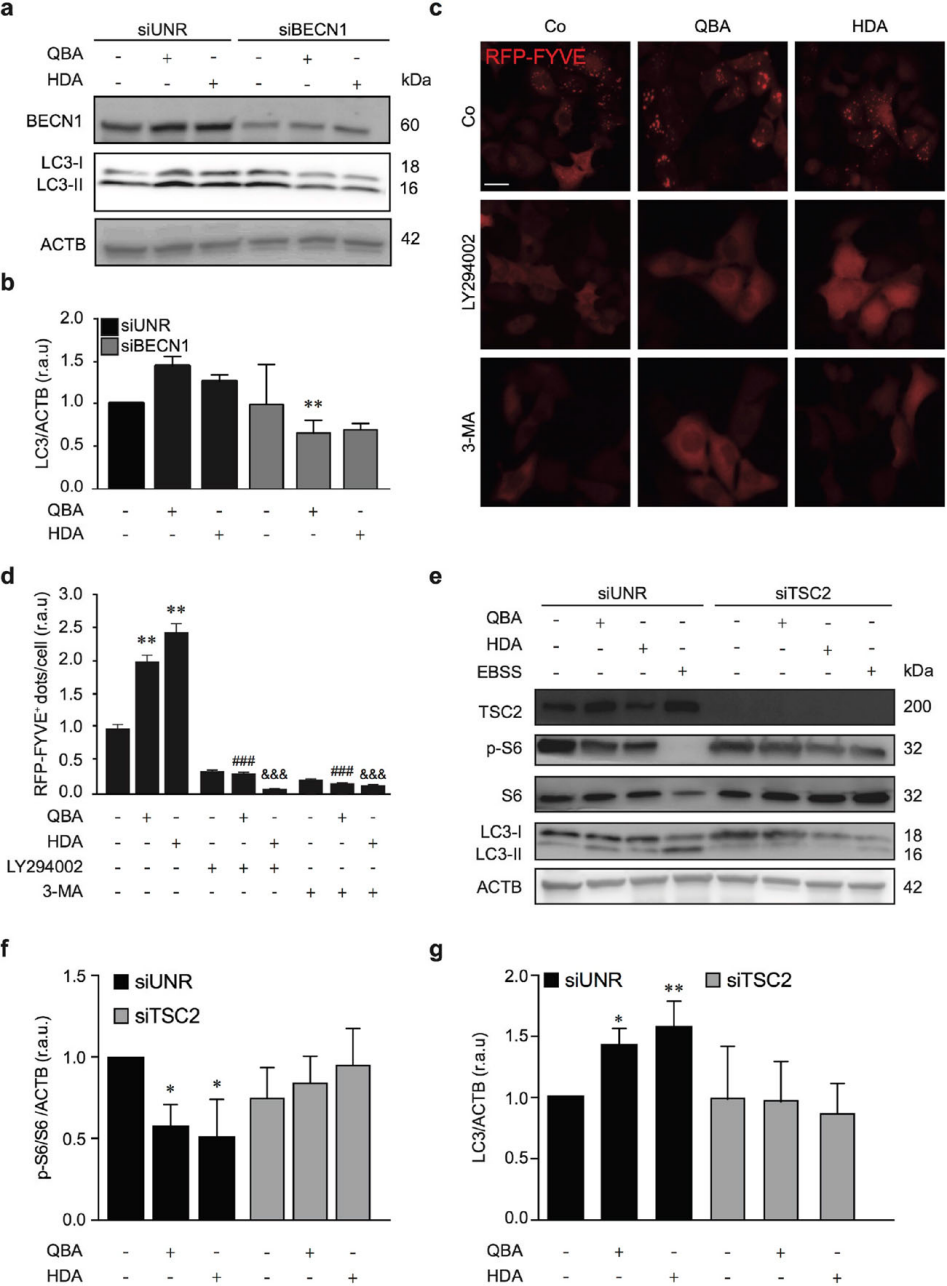
Upstream regulators of autophagy induced by the lipid fraction of RJ. (a, e) SH-SY5Y cells were transfected with siUNR or with siRNA specific for BECN1 (siBECN1) or TSC2 (siTSC2) for 48 h, and either kept in control conditions (Co) or EBSS (e) or treated with 50 μM QBA or 50 μM HDA for 4 h (a, e). Thereafter, LC3 lipidation (a, e) and S6 phosphorylation (e) were assessed by immunoblotting. BECN1, TSC2, S6, and ACTB levels were monitored as a genotype control and to ensure equal loading of lanes, respectively. Densitometry was employed to quantify the abundance of LC3-II (b, g) and p-S6 (f). *p <0.05, **p <0.01 versus untreated cells and non-significant at p >0.05 in siTSC2. (c, d) RFP-FYVE^+^ expressing U2Os cells were cultured in control conditions (Co) or treated with 50 μM QBA or 50 μM HDA alone or in combination with 10 μM LY294002 or 10 mM 3-MA. Four hours later, RFP-FYVE^+^ dots were quantified by fluorescence microscopy. Data are normalized as the means ± SD of at least 2 independent experiments (**p <0.01 vs. control cells, ^###^p <0.001 versus QBA-treated cells, ^&&&^p <0.001 versus HDA-treated cells). Scale bar = 10 μm. r.a.u: relative arbitrary units

As shown in Fig. 2a and b, the depletion of BECN1 significantly reduced the lipidation of LC3 by QBA treatment in SH-SY5Y cells. Similar results were observed in HDA-treated cells. To confirm the involvement of the BECN1 complex in the activation of autophagy induced by QBA, we assessed the production of PI3P mediated by PI3K-III using U2OS cells stably expressing RFP-tagged FYVE. This domain recognizes and binds to PI3P (Yakhine-Diop et al. 2017). Figure 2c, d show that QBA treatment increased RFP-FYVE puncta compared to control cells. Similar to QBA, HDA treatment enhanced the number of FYVE^+^ dots. However, the inhibition of PI3K activity using LY294002 and 3-MA abolished the accumulation of FYVE^+^ dots in QBA-treated cells. The same result was observed in HDA-treated cells.

mTOR negatively regulates autophagy activity in response to nutrient depletion (Liu and Sabatini 2020). Similar to EBSS, QBA treatment significantly diminished the phosphorylation levels of S6 kinase (Fig. S2a, b) and S6 protein (Fig. 2e–g) (downstream targets of mTOR), which are indicators of mTOR inhibition. In fact, the activation of mTOR kinase through the downregulation of tuberous sclerosis complex 2 (TSC2) increased the phosphorylation levels of S6 kinase and S6 protein, and diminished LC3 lipidation in QBA-treated cells, thereby inhibiting autophagic function. Taken together, these results demonstrated that QBA promotes autophagy not only by activating PI3K-III but also by suppressing mTOR activity.

### QBA induces SIRT1 nuclear translocation and deacetylation of ATG proteins

**Fig. 3:**
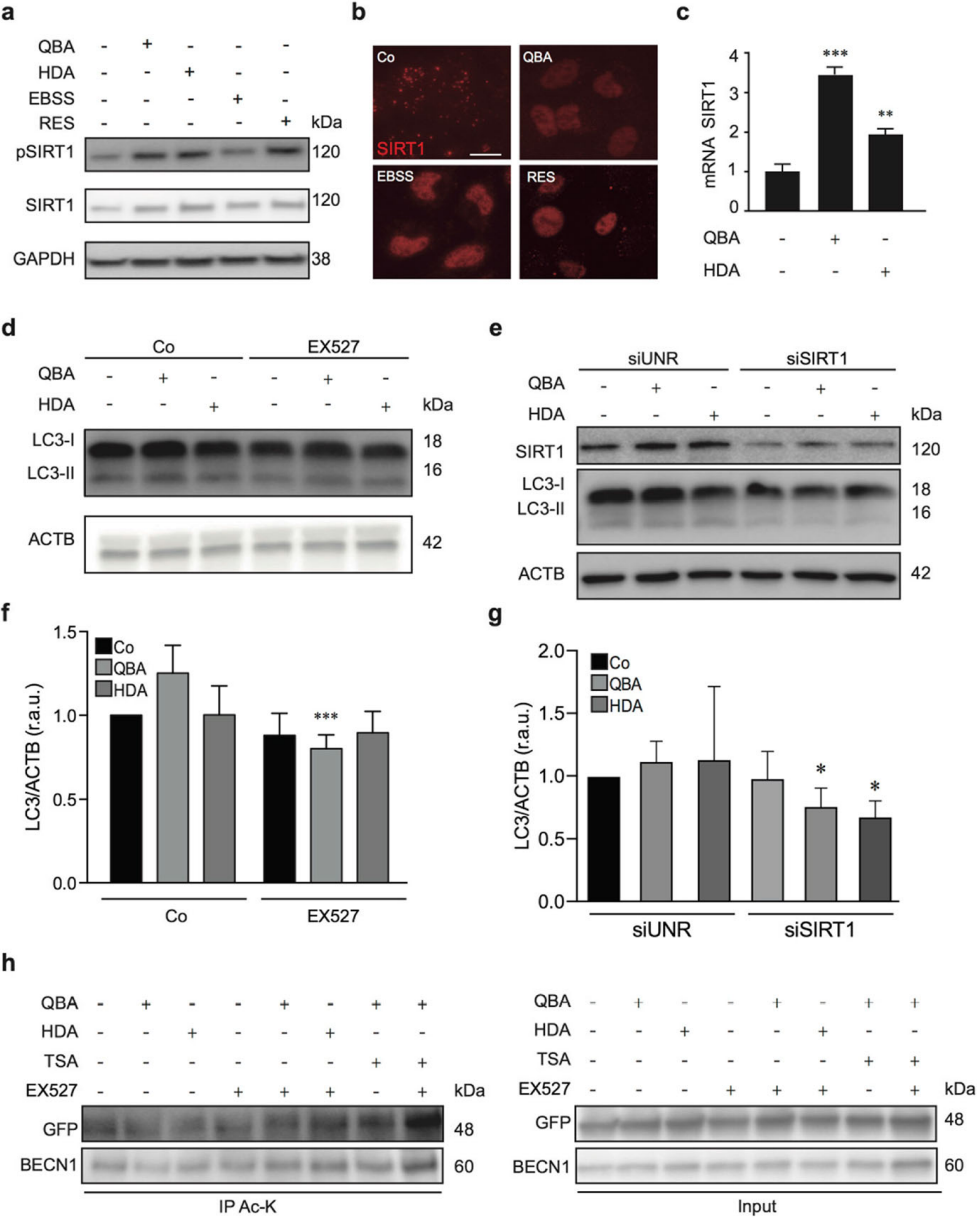
Implication of SIRT1 in QBA-induced autophagy. (a, b) SH-SY5Y cells were cultured in control conditions (Co), maintained in EBSS medium, exposed to 2 μM resveratrol (RES) or 50 μM QBA or 50 μΜ 10-HDA (HDA) for 4 h. Thereafter, cells were processed for the assessment of SIRT1 phosphorylation (Ser 47) (a) or SIRT1 localization detected by immunofluorescence (b). Scale bar = 10 μm. (c) SH-SY5Y cells were maintained in control conditions (Co), 50 μM QBA, or 50 μM HDA for 4 h. Thereafter, cells were processed and the expression of SIRT1 mRNA was examined by quantitative real-time PCR. **p <0.01, ***p <0.001 versus control cells. (d) SH-SY5Y cells were cultured in control conditions (Co) or treated with 50 μM QBA or 50 μM 10-HDA (HDA), alone or combined with 2 μM EX527 for 4 h. (e) SH-SY5Y cells were transfected with a control siRNA (siUNR) or with siRNAs targeting SIRT1 (siSIRT1) for 48 h and either maintained in control conditions (Co) or treated with 50 μM QBA or 50 μM 10-HDA for 4 h. Thereafter, cells were processed for the assessment of LC3 lipidation (d, e). ACTB levels were monitored to ensure equal loading of lanes and the densitometry was employed to quantify the abundance of lipidated LC3 (LC3-II) (f, g). Data are normalized as the means ± SD of at least three independent experiments (***p <0.001 vs. QBA-treated cells) (f), (*p <0.05 versus QBA-treated siUNR cells or HDA-treated siUNR cells) (g). r.a.u: relative arbitrary units. (h) H4-GFP-LC3 cells were cultured in control conditions (Co) or treated with 50 μM QBA or 50 μM HDA, alone or combined with 2 μM EX527 for 4 h. 1 μM TSA alone or combined with 2 μM EX527 was used as a control of protein acetylation Thereafter, LC3 and BECN1 were immunoprecipitated from cell lysates with anti-acetyl-lysine antibody (Ac-K) and analyzed by immunoblot using anti-GFP and anti-BECN1 antibodies.

Previous studies show that QBA is a class I and II histone deacetylase (HDAC) inhibitor (Spannhoff et al. 2011). We analyze the role of this fatty acid in the acetylation of autophagy-related proteins through SIRT1 activation. QBA and RES enhanced the phosphorylation of SIRT1 at serine 47 and promoted its translocation to the nucleus (Fig. 3a, b). Moreover, QBA remarkably increased mRNA *SIRT1* levels (Fig. 3c). To confirm the role of SIRT1 in QBA-induced autophagy, we inhibited its activity using a specific inhibitor (EX527) or by performing a gene silencing experiment (SIRT1-specific siRNA) in SH-SY5Y cells. As shown in Fig. 3d–g, the inhibition of SIRT1 significantly reduced the levels of LC3-II compared to control cells (siUNR). Similar results were observed in cells treated with HDA. Next, we focused on determining the mechanisms by which SIRT1 promotes QBA-induced autophagy. The analysis of acetylated protein levels showed that QBA treatment enhanced the levels of acetylated lysine proteins compared to control cells and HDA-treated cells (Fig. S3a, b). Next, we performed immunoprecipitation assays with an acetylated-lysine antibody in H4 cells stably expressing GFP-LC3. The acetylation levels of LC3 and BECN1 were detected by Western blotting by using an anti-GFP and BECN1 antibodies, respectively. We observed that LC3 and BECN1 were deacetylated in QBA-induced autophagy, and this effect was suppressed by the inhibition of SIRT1 (Fig. 3h and fig. S3c). Similar to QBA, HDA treatment reduced the acetylation levels of LC3; however, it did not promote BECN1 deacetylation.

These findings suggested that QBA induces autophagy through deacetylation of LC3 and BECN1 proteins mediated by SIRT1 activation. Therefore, we found that QBA and HDA induced autophagy in vitro through different signaling pathways. Based on these results for these two fatty acids, we selected QBA for its capacity to induce autophagy through SIRT1-mediated deacetylation of LC3 and BECN1.

### QBA induces autophagy in vivo

**Fig. 4:**
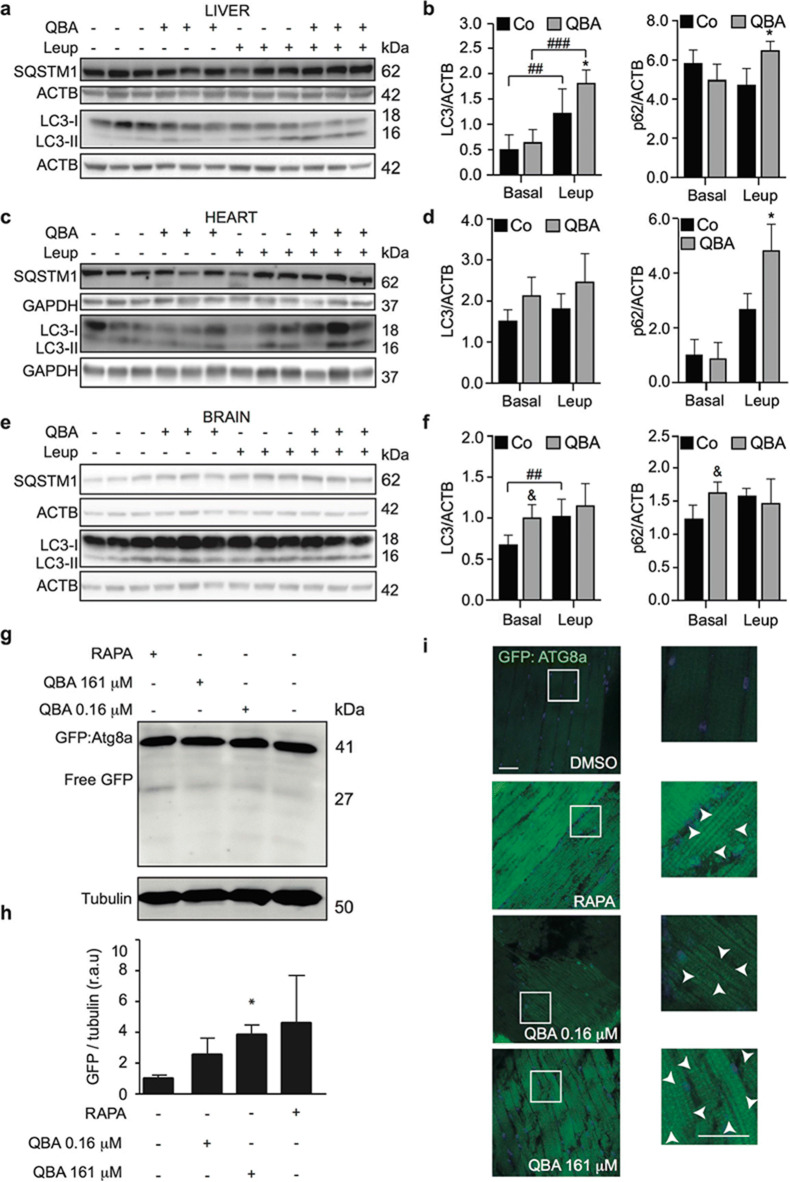
Analysis of QBA-induced autophagy in vivo. (a–f) ICR mice were injected i.p. with vehicle alone or with 10 mg/kg QBA. Two hours later, mice received vehicle or 15 mg/kg leupeptin (Leup). After 2 h, animals were euthanatized and LC3 lipidation was assessed by immunoblotting in the indicated tissues. ACTB and GAPDH levels were monitored to ensure equal loading of lanes, and densitometry was employed to quantify the abundance of lipidated LC3 (LC3-II) and p62. Data are the means ± SD of at least 3 mice in liver and heart, and at least 5 mice in brain (^##^p <0.01, ^###^p <0.001 vs. basal conditions) (*p <0.05 vs.leupeptin-treated mice) (^&^p <0.05 vs. untreated mice). (g, h) *Drosophila* Mhc-Gal4 >UAS-GFP:Atg8a line were collected in tubes containing food supplemented, with QBA (0.16–161 μM), rapamycin (RAPA, 50 μM), or DMSO as vehicle. Thereafter, GFP immunodetection was performed by Western blot. Tubulin levels were monitored to ensure equal loading of lanes (g), and densitometry was employed to quantify the abundance of GFP-Atg8a (h). Data are the means ± SD of 3 independent experiments (*p <0.05 vs. untreated flies). (i) Confocal fluorescence images of GFP immunostaining (green) in fly thoraces are shown. Nuclei were counterstained with DAPI (blue). Scale bar = 20 μM.

To analyze whether this fatty acid induces autophagy in vivo, ICR mice were injected with QBA or the saturated fatty acid HDA at a concentration of 10 mg/kg for 4 h. Several tissues were collected, and the analysis of LC3-II levels was monitored by immunoblotting. Figure S4a, b shows that QBA treatment remarkably induced the conversion of LC3-I to LC3-II in liver and heart tissues. Similar results were observed in HDA-treated mice or in response to nutrient deprivation. Moreover, the analysis of brain tissue showed that QBA significantly enhanced the lipidation of LC3 compare to untreated mice (Fig. 4e, f and S4c). To confirm these results, we analyzed autophagy flux in vivo after the administration of leupeptin, a protease inhibitor. LC3 only increased in liver while p62 significantly enhanced in liver and heart (Fig. 4a-d). Surprisingly, there were no changes in brain. These data indicates that QBA enhances autophagy flux in liver and heart but not in brain.

Moreover, we analyzed whether QBA triggered autophagy in *Drosophila melanogaster*. To this end, transgenic adult *Drosophila* that express GFP:Atg8a were treated with QBA and rapamycin. As shown in Fig. 4g and h, the analysis of GFP-Atg8 by immunoblotting indicated a significant increase of the free-GFP signal in QBA-treated flies compared to control. These results were confirmed by confocal studies. Similar to rapamycin, QBA-treated flies showed an accumulation of GFP:Atg8a puncta compared to untreated flies (Fig. 4i). These results confirmed that QBA induces autophagy in mice and flies.

### Inhibition of QBA-induced autophagy enhances 6-OHDA-induced toxicity

**Fig. 5:**
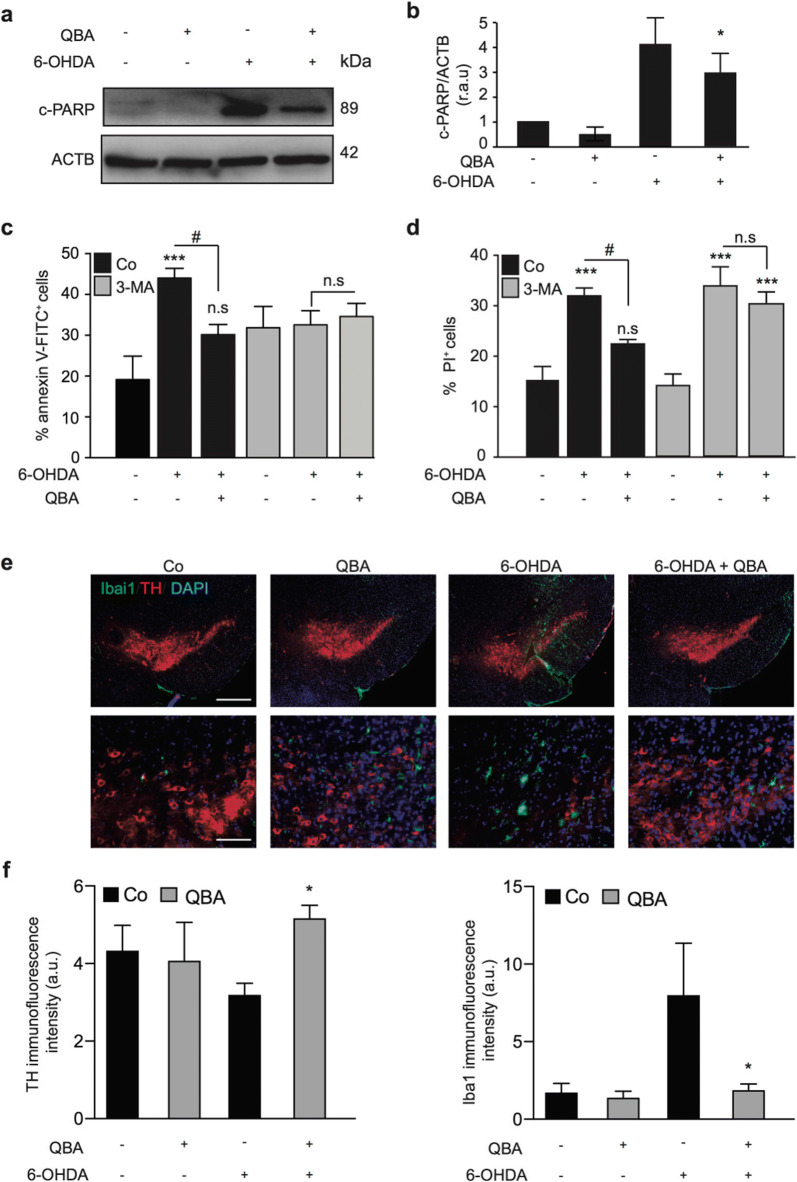
QBA decreases 6-OHDA-mediated toxicity in an autophagy-dependent manner*.* (a–d) SH-SY5Y cells were cultured in control conditions (Co), or pre-treated with 50 μM QBA for 24h, followed by the treatment with 35 μM 6-hydroxydopamine (6-OHDA) alone (a, b) or combined with 10 mM 3-methyladenine (3-MA) for 18 h (c, d). Cleaved PARP (c-PARP) was assessed by immunoblotting. ACTB was used as a loading control (a) and densitometry was employed to quantify the abundance of c-PARP (b). *p <0.05 compare with 6-OHDA-treated cells (c, d). The percentage of Annexin V-FITC-positive cells (c) and PI-positive cells (d) were evaluated by flow cytometry (n=10000 events). Columns indicate means ± SD. Symbols indicate significant, ***p˂0.001, and non-significant (n.s.) compared with the respective untreated groups, ^#^ p<0.05, compare with 6-OHDA-treated cells (c,d). (e) QBA treatment reduces microglial activation in the SNpc with 6-OHDA injections*.* Mice were injected with PBS (Co) or QBA alone or in combination with 6-OHDA. Animals were sacrificed, and the brains were processed by immunofluorescence. Immunostaining showing the expression of the microglial marker Ibai1 (green), tyrosine hydroxylase (TH, red), and DAPI (blue) in the *SNpc* (e). Scale bar= 200 μm. The quantification of TH and Iba1 intensity is shown in (f). Symbols indicate significant *p <0.05 compared with 6-OHDA-treated mice.

Several studies have demonstrated the pharmacological properties of QBA in animal models (Hattori et al. 2007; Honda et al. 2015; Watadani et al. 2017; Weiser et al. 2017). We analyzed whether QBA-induced autophagy exerts cytoprotective effects in a neurodegenerative model. To perform this experiment, we first examined the effect of QBA in SH-SY5Y cells treated with the neurotoxin 6-hydroxy-dopamine (6-OHDA). The levels of poly (ADP-ribose) polymerase 1(PARP1), a substrate of caspase 3, was assessed by Western blotting. Our results showed that the pre-treatment of QBA notably reduced the levels of cleaved PARP1 (Fig. 5a, b) in 6-OHDA-treated cells. This decrease correlated with a reduced level of cleaved caspase 3 after QBA pretreatment in N2a cells (Fig. S5a, b). By flow cytometry, we also observed that QBA treatment decreased the cell death induced by 6-OHDA (Fig. 5c, d and fig. S5c, d). These results suggest that QBA treatment partially reduces the toxicity of 6-OHDA. To confirm that this effect was autophagy-dependent, cells treated with 6-OHDA were exposed to QBA in the presence or not of 3-MA and we remarked that the cytoprotective effect of QBA was reduced after inhibition of autophagy in SH-SY5Y (Fig. 5c, d) and N2a cells (Fig. S5c, d). We also investigated the effect of QBA treatment in a PD mouse model. Mice were injected in the *substantia nigra pars compacta* (*SNpc*) with QBA and/or 6-OHDA, and later brain tissue was prepared for immunohistochemical analysis of cell death and the inflammation response by analyzing the expression of tyrosine hydroxylase (TH) (an enzyme involved in dopamine production) as a marker of dopaminergic cells, Iba1, to label microglial cells and DAPI for nuclear staining. Triple immunofluorescence representative images (Fig. 5e and f) showed that QBA injection significantly reduced the inflammatory response and cell death induced by 6-OHDA intracranial injection. These findings suggest that QBA is able to trigger autophagy and to prevent 6-OHDA-induced toxicity in vitro as well as in vivo.

### QBA-induced autophagy promotes longevity in D. melanogaster

**Fig. 6:**
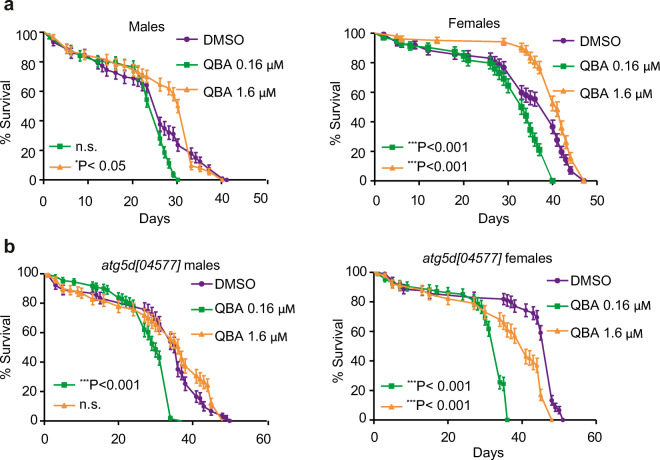
QBA enhances longevity in flies. (a, b) *w*^*1118*^ and *Atg5d [04577] flies* were maintained with standard nutritive medium supplemented with QBA (0.16–161 μM) or DMSO as vehicle. Survival curves were obtained using the Kaplan-Meier method. P-values were calculated with the Gehan-Breslow Wilcoxon Test. Symbols indicate significant, *p <0.05, ***p˂0.001 and non-significant (n.s.) compared with the respective untreated.

It has been shown that the induction of autophagy is required for longevity extension (Melendez et al. 2003; Nakamura and Yoshimori 2018; Pyo et al. 2013; Toth et al. 2008). Several studies have demonstrated that RJ promotes longevity in several animal models (Honda et al. 2011; Inoue et al. 2003; Xin et al. 2016). Moreover, QBA extends the lifespan in *C. elegans* (Honda et al. 2015). Taking this background into account and our results, we hypothesized that QBA can promote longevity through autophagy induction. To confirm this hypothesis, we performed a survival assay in *Drosophila melanogaster* and we observed that the flies exposed to QBA (1.6 μM) showed a significant increase in lifespan compared to unexposed flies (Fig. 6a). Surprisingly, 0.16 μM QBA significantly reduced lifespan in healthy female flies compared to DMSO. Next, we analyzed whether the anti-aging effect of QBA depends on autophagy using a *D. melanogaster* strain lacking *Atg5*. As shown in Fig. 6b, the increase of longevity mediated by QBA was suppressed in *Atg5 ko* flies compared to control flies. Indeed, QBA significantly decreased lifespan compared to the vehicle (DMSO) in both male (0.16 μM QBA) and female (0.16/1.6 μM QBA) *ATG5 ko* flies. The data suggests that, when QBA is administered to autophagy-deficient flies, the agent accelerates death. Therefore, our results showed that the QBA-mediated longevity extension is autophagy-dependent.

## Discussion

QBA, the major lipid of RJ, has attracted increasing attention due to its beneficial effects on health. Several studies have reported its therapeutic properties; however, the precise molecular mechanisms by which QBA exerts these effects have not been elucidated (Izuta et al. 2009; Peng et al. 2017; Sugiyama et al. 2012; Yang et al. 2018; You et al. 2019a). The present study now provides compelling evidences that QBA is a novel autophagy inducer that promotes its health-promoting effects through autophagy activation.

Autophagy is an evolutionarily conserved self-degradative mechanism responsible for the removal of aberrant misfolded proteins and damaged organelles of all eukaryotic cells. Accumulated evidence has revealed that autophagy is essential for the healthy aging of neurons (Menzies et al. 2017; Rubinsztein et al. 2012). In fact, impaired autophagy is associated with neurodegeneration (Rubinsztein et al. 2012). The discovery that the activation of autophagy by nutritional, pharmacological, or genetic interventions can reduce age-related diseases and/or extend longevity has increased scientific interest in identifying novel autophagy inducers that function as a potential therapeutic strategy to ameliorate neurodegenerative disorders. In this sense, many novel autophagy enhancers have been isolated from natural products (Eisenberg et al. 2009; Kim et al. 2013; Morselli et al. 2010; Ryu et al. 2016; Sarkar et al. 2007; Shakeri et al. 2019). RJ is a very interesting natural product because it contains health- and longevity-promoting factors that enhance the overall health, fertility, and longevity of the queen bees (Spannhoff et al. 2011; Yang et al. 2012). In addition to its beneficial effects, it has been described that RJ extends the lifespan of nematodes, flies, and mice (Honda et al. 2011; Inoue et al. 2003; Xin et al. 2016). Most studies with RJ suggest that bioactive compounds that include fatty acids and proteins might be responsible for its beneficial health effects (Fratini et al. 2016; Kunugi and Mohammed Ali 2019; Li et al. 2013; Wan et al. 2018). In this study, we have identified two fatty acids isolated from RJ that function as novel autophagic enhancers (QBA and HDA); however, QBA was more efficient. We found that QBA triggers autophagy in neuronal cell lines and animal models (Fig. 1 and fig. 4). The activation of autophagy mediated by QBA is BECN1 and mTOR-dependent (Fig. 2). Moreover, QBA deacetylates ATG proteins through SIRT1 activation (Fig. 3). Interestingly, QBA-mediated autophagy exerts neuroprotective effects in models of 6-OHDA-mediated toxicity (Fig. 5) and extends the lifespan of *D. melanogaster* (Fig. 6).

Despite the numerous studies that reported the therapeutic properties promoted by QBA, the mechanism of action of this fatty acid is currently unknown. Previous studies have reported that several fatty acids trigger autophagy in multiple cell types (Chen et al. 2018; Gwon et al. 2017; Niso-Santano et al. 2015). In this study, we analyzed whether the health beneficial properties of QBA and its lifespan extension effects occur through the activation of autophagy. Remarkably, QBA enhances the recruitment of LC3 to the autophagosome membrane, increases lysosomal activity, and promotes the degradation of long-lived proteins in neuronal cell lines. One of the first events in autophagy is autophagosome formation. In fact, the PI3K-III complex regulates the size and rate of production of autophagosomes through PI3P synthesis in response to nutrient starvation (McKnight and Zhenyu 2013). Our results showed that QBA regulates the initiation step of autophagosome biogenesis through PI3P synthesis and BECN1. Although PI3K-III is responsible for most PI3P synthesis, mammalian cells can produce PI3P through alternative mechanisms (Vicinanza et al. 2015). However, in our model, the production of PI3P depends on PI3K-III because LY294002 and 3-MA treatment (an inhibitor of PI3K-III) decreased FYVE-induced QBA.

mTOR is a major upstream signaling pathway that induces autophagy in mammalian cells (Laplante and Sabatini 2012). This protein regulates autophagy in response to nutrient availability (Gonzalez and Hall 2017). mTOR activity is regulated by the PI3K/AKT signaling pathway and AMPK activation (Gwinn et al. 2008; Sarbassov et al. 2005). Previous studies have demonstrated that QBA can alleviate neuroinflammation by the activation of the AMPK pathway and the downstream PI3K/AKT pathway (You et al. 2019a). Our results confirmed the activation of AMPK in QBA-treated cells (data not shown) and the inactivation of mTOR. Moreover, the activation of mTOR inhibits QBA-induced autophagy.

In parallel, QBA is a novel SIRT1 activator and induces autophagy through the deacetylation of ATG6 and ATG8 proteins. SIRT1 is (a NAD-dependent histone deacetylase) a class III histone deacetylase that functions as a key metabolic/energy sensor and promotes autophagic responses by the deacetylation of several ATG proteins under nutrient deprivation conditions (Cohen et al. 2004; Lee et al. 2008). It has been reported that QBA is an inhibitor of histone deacetylase (Spannhoff et al. 2011). Consistent with this, QBA treatment increased protein acetylation levels and stimulated the phosphorylation of SIRT1 (Fig. 3). Consequently, its translocation to the nucleus enhanced the deacetylation of key ATG proteins such as LC3 and BECN1. All these effects were prevented by the inhibition of SIRT1. It has been reported that autophagy is activated under nutrient stress conditions through SIRT1-Forkhead protein O 1 (FOXO1)–dependent mechanisms (Brunet et al. 2004; Nemoto et al. 2004). FOXO1 activity is regulated by its acetylation status. The acetylation of FOXO-1 can lead to inhibition of autophagic responses, while its deacetylation by SIRT1 activates autophagy (Brunet et al. 2004; Nemoto et al. 2004). A recent study demonstrated that QBA improved lipopolysaccharide-induced neuroinflammation in rodents via autophagy induction (You et al. 2019b). Given that FOXO1 is implicated in QBA-mediated autophagy, we surmise that SIRT1 can modulate its anti-inflammatory effect through the induction of autophagy.

CR is one of the most effective strategies to improve health and survival in several animal models through autophagy activation. Notably, CR regulates autophagy through nutrient-sensing signaling proteins including mTOR, AMPK, and SIRT1 (Cohen et al. 2004; Efeyan et al. 2015; Kim et al. 2011). Given that QBA activates the same signaling pathways, it could be considered as a CRM compound. In addition, previous studies with RJ or its bioactive component QBA have demonstrated its effect on longevity in several species (Honda et al. 2015; Honda et al. 2011; Inoue et al. 2003; Xin et al. 2016). In our work, we showed that QBA treatment enhances the lifespan of *D. melanogaster*, and its effect depends on autophagy activation. This result is consistent with a study showing that QBA delays aging in *C. elegans* in a TOR-dependent manner (Honda et al. 2015). Additionally, the administration of QBA in vivo promotes neurogenesis and neuronal health (Hattori et al. 2007). However, few studies have analyzed the effect of QBA in neurodegenerative disease. Our results showed that QBA enhances p62 in tissue samples being more significant in brain. This increase could be attributed to QBA-induced mRNA expression level (data not shown). The analysis of autophagy flux in the brain did not show statistical differences. Although leupeptin crosses the BBB, multiple cell types exist in the brain which may show distinct autophagy responses. In fact, previous studies have already demonstrated that starvation does not provoke significant changes in brain (Mizushima et al. 2004). Moreover, the injection of QBA into *SNpc* significantly reduced the death of dopaminergic neurons as well as the inflammatory response observed after intracranial injection of 6-OHDA. Therefore, QBA exerts protective effects against neurodegeneration in vitro and in a PD-mouse model. The inhibition of autophagy by 3-MA increased the cytotoxic effect of 6-OHDA in vitro. Therefore, the autophagy-stimulating activity of QBA is associated with beneficial health effects in a PD model.

In summary, to the best of our knowledge, this study is the first to classify the two major fatty acid of Royal Jelly, QBA, and HDA, as novel autophagy inducers. Interestingly, QBA is more efficient and it acts on the same signaling pathways as CR. Most importantly, QBA-induced autophagy is able to exert a neuroprotective effect in PD models and reproduces the longevity benefits of CR in *D. melanogaster*, suggesting that this fatty acid may be a new potential CRM.

## Materials and methods

### Cell lines and culture conditions

Human neuroglioma H4 cells stably expressing GFP-LC3 and human osteosarcoma U2OS cells stably expressing RFP-FYVE (Zhang et al. 2007) were maintained in Dulbecco’s modified Eagle’s medium (DMEM) high glucose, pyruvate (Gibco, D6546-500 mL) supplemented with 10% heat-inactivated fetal bovine serum (SIGMA, F7524 500 mL), 10 mM HEPES buffer (GIBCO 15630056), 2 mL of penicillin (10 U/ml) and streptomycin (100 μg/mL) (GIBCO, SV30010), and 500 μg/mL G418. Human dopaminergic neuroblastoma SH-SY5Y, stable ATG5 knockdown SH-SY5Y, and mouse neuroblastoma Neuro-2a (N2a) cells were collected in RPMI medium (HyClone 1640) supplemented with 10% heat-inactivated fetal bovine serum (SIGMA, F7524-500 mL), 200 mM l-glutamine, and 0.04 mg/mL gentamicin (Gibco 15750037). *Wild-type (WT) and atg5 knockout* embryonic mouse fibroblasts (MEFs) were maintained in DMEM (SIGMA, D6546-500 mL) supplemented with 10% heat-inactivated BGS (HyClone, sh30541.3), 200 mM l-glutamine, and 10 U/mL penicillin and streptomycin (100 μg/mL) (GIBCO, SV30010). The cells were cultured in 75 or 150 tissue culture flasks (Thermo Scientific™ 130190) and maintained in 5% CO_2_/95% air at 37°C under saturating humidity. Cells were passaged every 24–48 h.

### Reagents and chemical treatment

Cells grown to 75% confluency were seeded in 6-, 24-, or 96-well plates. After 24 h, cells were treated with QBA (#cay10976-500 mg, Vitro) and HDA (#379700, Sigma-Aldrich) at different concentrations for 2–4 h. We used serum-free Earle’s balanced solution salt (EBSS, #E2888, Sigma-Aldrich), rapamycin (RAPA, 1 μM, #R-5000, LcLabs), or resveratrol (RES, 2 μM, #1418, Tocris) for 4 h to induce autophagy and 3-methyladenine (3-MA) for 1 h (10 mM, #m9281, Sigma-Aldrich), bafilomycin A1 (BAF.A1,100 nM, #b1080, LcLabs), or LY294002 (10 μM, #1130, Biogen) for 4 h to inhibit autophagy. Cells were treated with EX527 for 4 h (2 μM, #E7034, Sigma-Aldrich) to inhibit the protein deacetylase SIRT. 6-Hydroxydopamine (6-OHDA, 35 μM, #H116, Sigma-Aldrich) was added to the medium for 20 h and Trichostatin A (TSA, 1 μM, #T8552, Sigma-Aldrich) was added to the medium for 4 h to the inhibition of class I and II HDACs.

### Protein isolation and Western blotting analysis

To isolate proteins, cells were collected and lysed in a buffer containing 0.5% NP-40, 100 mM Tris HCl, 300 mM NaCl, 1% SDS, and protease (#11836170001, Roche) and phosphatase inhibitors (PhosSTOP (#4900837001, Roche). The tissue samples were lysed with lysis buffer containing 150 mM NaCl, 7.5 mM Tris HCl pH 7.5, 1 mM EDTA and complemented with protease and phosphatase inhibitors. Total protein concentration was measured by the bicinchoninic acid assay (#B9643-1L, Sigma-Aldrich) according to the manufacturer’s instructions. Protein samples (20 μg for each samples) were subjected to an electrophoresis on 12% Tris-Glycine Mini-PROTEAN TGXTM gels (10 or 15 well, Bio-Rad) or Criterion TGX Gels (18 well, Bio-Rad) and electrotransferred onto PVDF membranes (#1620177, Bio-Rad). Later, membranes were blocked (1h at RT) with 10% (w/v) fat-free milk in Tris-buffered saline (10 mM Tris/HCl pH 7.5, 150 mM NaCl) containing 0.2% Tween-20 (#P5927, Sigma-Aldrich) (TBST). Membranes were washed with TBST 1X and incubated overnight at 4°C with the corresponding primary antibodies specific to AcK (#9441, Cell Signaling Technologies), ATG5 (#1672630s Cell Signaling Technologies), ACTB (#ab49900, Abcam), BECN1 (#sc-11427, clone H-300, Santa Cruz Biotechnology Inc), Cathepsin B (#ab58802, Abcam), cleaved caspase-3 (Asp175, #9661, Cell Signaling Technologies), cleaved PARP (Asp214, #9541, Cell Signaling Technologies), GAPDH (NG1740950, Millipore), GFP (#29565, Cell Signaling Technologies), LAMP2 (sc18822, Santa Cruz Biotechnology Inc.), LC3-B (#L7543, Sigma-Aldrich), PARP (#9542, Cell Signaling Technologies), p62/SQSTM1 (#H00008878-MO1, Abnova), pS6 *ribosomal protein* (Ser235/236, #4858, Cell Signaling Technologies), pSIRT1 (Ser 47, #2314, Cell Signaling Technologies), pS6 kinase (Thr389, #9205 Cell Signaling Technologies), S6 *ribosomal protein* (#2317, Cell Signaling Technologies), S6 kinase (#9202, Cell Signaling Technologies), SIRT1 (#9475, Cell Signaling Technologies), and TSC2 (#3612, Cell Signaling Technologies). After several washing steps in TBST 1X, the membranes were incubated with their respective HRP-conjugated secondary antibodies (1:10000) (#170–6515 and #170-5047, Bio-Rad, for rabbit and mouse antibodies, respectively) for 1h at room temperature. Immunoreactive bands were visualized with ECL substrate (ThermoScientific, 32106) and chemiluminescence images were captured by an Amersham Imager 600 (GE Heathcare). Band intensities were quantified using ImageJ software (NIH), establishing ACTB, GAPDH, or tubulin protein levels as a loading control.

### RNA interference

Once cells reached 50% confluence, cells were transfected with siRNAs targeting BECN1 (siBECN1, 50 UUCCGUAAGGAACAAGUCGGdTdT-30), SIRT1 (siSIRT1, #AM16708, Ambion), and TSC2 (siTSC2, #5269468, Invitrogen-Life) using HiPerfect Transfection Reagent (#509301704, Qiagen) according to the manufacturer’s instructions. An irrelevant siRNA duplex (siUNR (Ambion, L/N 1602012)) was employed as a negative control. Forty-eight hours later, transfection efficiency was determined by Western blotting.

### Expression analysis by real-time RT-PCR

Total RNA was extracted using an RNeasy mini kit (#74104, Qiagen). Genomic DNA contamination was eliminated from total RNA samples using an RNase-Free DNase kit (#AMPD1-1KT, Sigma-Aldrich). RNA purity and quantity were checked with a NanoDrop. A total of 500 ng of total RNA was reverse transcribed into complementary DNA (cDNA) via QuantiTect reverse transcription (#205311, Qiagen) in a 20-μL reaction according to the manufacturer’s protocol. The resulting cDNA was amplified by real-time RT-PCR and SIRT mRNA expression was measured by a KAPA SYBR® Fast kit (#KK4601, Kapa Biosystems) using the following primers from IDT®: GAPDH (5′-AGCCACATCGCTGAGACA-3′) and SIRT1(5′-TGCGGGAATCCAAAGGATAATTCAGTGTC-3′). The expression of the housekeeping gene GAPDH was used to normalize the result. The expression levels were determined by the 2(^−ΔΔCt^) ratio.

### Automated fluorescence microscopy

GFP-LC3 H4 and RFP-FYVE U2Os cells were plated on 96-well TC-treated Imaging Microplates (BD Falcon, Sparks, MD, USA) and cultured for 24 h. After treatments, cells were washed in PBS, fixed (for 15 min at RT) with 4% PFA, and stained with 2 μM Hoechst 33342 (#B2261, Sigma-Aldrich). After several washing steps with PBS (#mb18201, Nzytech), cells were permeabilized with 0.1% Triton X-100 (#T9284, Sigma-Aldrich) for 10 min RT and blocked (for 1 h at RT) with bovine serum albumin (BSA)/PBS solution (1 mg/mL). Thereafter, cells were incubated with the specific primary antibody overnight at 4°C followed by incubation with appropriate rabbit or mouse Alexa Fluor® 568 (Thermo Scientific, A11004) or 488 (Thermo Scientific, A11008)-conjugated secondary antibodies (for 1 h at RT). Image acquisition was done with a Cellsens IX83 molecular inverted microscope. The presence of cytoplasmic GFP-LC3^+^ and RFP-FYVE^+^ dots was assessed using Ifdotmeter® software (Rodriguez-Arribas et al. 2016).

### Confocal microscopic analysis

GFP-LC3 H4 cells were seeded in 96-well TC-treated Imaging Microplates (BD Falcon, Sparks, MD, USA) 24 h before stimulation. After experimental treatments, cells were fixed with 4% PFA supplemented with DAPI (ThermoFisher, P36966). Fluorescence images were visualized using an A1 confocal imaging system mounted on an inverted Eclipse Ti microscope (Nikon Corp., Tokyo, Japan A1). LC3 and lysotracker or SQSTM1 cytoplasmic colocalization data were analyzed with the JACoP plugin of ImageJ software with preprogrammed macros. At least 100 hundred cells were analyzed in every experiment.

### Immunoprecipitation assay

For acetylation analysis, whole-cell extracts were lysed with cell lysis reagent (CelLytic^TM^ M, c2978, Sigma-Aldrich) supplemented with 10% deacetylase inhibitor cocktail (#sc362323, Santa Cruz Biotechnology Inc., Santa Cruz, CA, USA) and 10 μM anacardic acid (#A7236, Sigma-Aldrich). Two hundred micrograms of protein was incubated with Dynabeads protein G (#1007D, Novex) for 90 min. Once the beads attached to the nonspecificities were discarded, samples were incubated with Ac-K antibody overnight at 4°C in agitation. Proteins were then reincubated with Dynabeads for 150 min. Immunoprecipitated proteins were washed three times with lysis buffer and eluted with Laemmli 2x and SDS loading buffer 5x for 1 min at 95 °C.

### Mouse experiments

ICR mice were kept under controlled conditions with food and water ad libitum. Four-week-old male animals were injected intraperitoneally with either 10 mg/kg QBA or 10 mg/kg HDA or an equivalent volume of vehicle. Four hours later, mice were euthanized. To induce autophagy, animals were starved overnight. To analyze autophagic flux, mice received an intraperitoneal injection of QBA, and 2 h later, mice were again injected intraperitoneally with 15 mg/kg leupeptin (in 150 mM NaCl) or an equivalent volume of vehicle and were sacrificed 2 h later. Thereafter, organs were collected and processed for immunoblotting detection of LC3 lipidation. All animal experiments were approved by the Committee for Animal Experimentation of the Universidad de Extremadura and were performed in accordance with its guidelines.

In 6-OHDA studies, C57BL/6 male mice, 2 to 3 months of age, were maintained on a 12-h light/dark cycle with access to food and water ad libitum. The animal protocols used in this study were performed in accordance with institutional guidelines (Institutional Animal Care and Use Committee guidelines) and approved by the “Ethics committee for Animal Experimentation” of the Instituto de Investigaciones Biomédicas (CSIC-UAM). Animals (four mice per experimental group) were anesthetized with a mixture of ketamine (60 mg/kg)/medetomidine (5 μg/kg) and placed in a stereotaxic apparatus (Koft Instruments, CA). Mice were then injected with either 1 μg of 6-OHDA dissolved in 2.5 μL of PBS or QBA (11 μg in 2.5 μL of PBS) or the same dose of 6-OHDA in combination with QBA (11 μg in 2.5 μL of PBS) into the right side of the SNpc at the following coordinates: posterior −3.2 mm, lateral +2.0 mm, and ventral +4.7 mm relative to Bregma and according to the atlas of Paxinos and Watson (Paxinos 1998). Parallel injections of 2.5 μL of PBS served as controls for the 6-OHDA-treated animals. To analyze the effect of autophagy inhibition, mice were anesthetized and injected with 6-OHDA (1 mg in 2.5 mL of PBS) or 6-OHDA in combination with QBA (11 μg in 2.5 μL of PBS) as described previously. After different treatments, mice were housed individually to recover and sacrificed 7 days after lesioning.

### Histology and immunohistochemistry

Mice were anesthetized and transcardially perfused with ice-cold PBS and 4% paraformaldehyde (PFA). Brains were harvested, postfixed in 4% PFA at 4°C overnight, cryoprotected in 30% sucrose, and stored at −80°C. Brain tissue was cut into 30-μm-thick sections using a cryostat (Cryocut 1900, Leica) and stored in a solution containing 30% ethylene glycol, 30% glycerol, and 40% phosphate buffer 0.1 M at −80°C until use. For immunohistochemistry, sections were permeabilized and blocked with 0.1% Triton X-100 and 3% goat serum in PBS for 1 h at room temperature followed by overnight incubation with primary antibodies against monoclonal mouse tyrosine hydroxylase (TH) (T2928, Sigma-Aldrich) and polyclonal rabbit Iba1 (011-27991, Wako). After several washing steps, all sections were incubated for 1 h at RT with a secondary Alexa-Fluor 488 goat anti-rabbit or Alexa-Fluor 647 goat anti-mouse antibodies (1:500, Jakcson ImmunoResearch) and washed, and nuclei were stained with DAPI for 15 min at room temperature. Finally, coverslips were mounted in Vectashield H-1000 (Vector Laboratories). Images were acquired under a fluorescence microscope (Nikon 90i).

### *D. melanogaster* studies

*w*^*1118*^, Mhc-Gal4 >UAS-GFP:Atg8a, *Atg5d[04577]* lines were obtained from the Bloomington Drosophila Stock Center (Indiana University, Bloomington, IN, USA). *Mhc-Gal4* flies were described previously (Marek et al. 2000). Flies were maintained at 25 °C with standard fly food. For oral administration of QBA, a maximum of 25 1-day-old adult flies was collected in tubes containing standard food supplemented with QBA (0.16–161 μM). Flies were transferred to tubes containing fresh food every 2–3 days. GFP (11814460001, Sigma-Aldrich) and α-tubulin (clone 12G10, Developmental Studies Hybridoma Bank) immunodetection in protein extracts and GFP:Atg8 in fly thoraces was performed as previously described (Bargiela et al. 2015).

### *D. melanogaster* lifespan analyses

A total of 90–100 newly emerged flies were collected in freshly prepared tubes containing standard nutritive medium with QBA (concentrations ranging from 0.16 to 161 μM), rapamycin (50 μM), or DMSO as vehicle. Males and females were kept in different tubes at 29°C. The number of dead flies was scored daily. Flies were transferred to new tubes twice a week. Survival curves were obtained using the Kaplan-Meier method and were statistically compared according to the Gehan-Breslow-Wilcoxon test (α=0.05).

### Flow cytometry assay

Cells were seeded in 24-well plates and cultured for 24 h. After experimental interventions, cells were collected in FACS tubes (51031, Enzo laboratories) and stained with Annexin V-FITC (Immunostep, ANXVF-200T) for 15 min at 37°C and after propidium iodide (0.1 mg/mL) (PI, Sigma-Aldrich, P4170) was added to each tube to detect either the percentage of PI- or Annexin-positive cells using a Beckman Coulter FC500-MPL.

### Degradation of long-lived proteins

Cells were seeded in 6-well plates and cultured for 24 h. After treatments, cells were maintained with 0.2 lCi/mL [14C]-valine (NEC291EU050UC, PerkinElmer) for 18 h at 37°C. After several washing steps in PBS (pH 7.4), the medium with the isotope-labeled amino acid was changed for a cell culture medium with l-valine (V0153, Sigma-Aldrich), first for 1h (prechase), and then in combination with several experimental treatments (chase) for 4 h. Thereafter, proteins were precipitated with 10% (v/v) trichloroacetic acid (TCA) (T9159, Sigma-Aldrich), separated from the soluble radioactive fraction by centrifugation at 6000 g for 20 min and then dissolved in 0.2 N NaOH. Finally, the long-lived protein degradation was measured by adding a small quantity of each sample into a scintillation liquid and measuring the radioactive activity with a liquid scintillation counter.

### Statistical analyses

Data are expressed as the mean ± SD of triplicate determinations. Experiments were repeated at least three times, yielding similar results. Unless otherwise indicated, comparisons among groups used unpaired Student’s test, one-way ANOVA, or two-way ANOVA with the Sidak or Tukey posttest.

## Supplementary Information

Fig S1 **Lipid fraction of RJ induces autophagy*.*** (a) U251 cells were cultured in control conditions (Co), incubated with EBSS medium, or treated with QBA (10, 25, 50, 100, 250, 500 and 1000 μM) for 4 h followed by the assessment of LC3 lipidation by western blot. ACTB levels were used as a loading control. (b) U251 cells were cultured in control conditions (Co), incubated with 10 nM rapamycin (RAPA), or treated with QBA (250 and 500 μM) alone or in combination with 100 nM BAF.A1 for 4 h followed by the assessment of LC3 lipidation by western blot. ACTB levels were used as a loading control. (c) H4 cells were cultured in control conditions (Co), incubated with 10 nM rapamycin (RAPA), or treated with 50 μM QBA or 50 μM HDA alone or in combination with 100 nM BAF.A1 for 4h followed by the assessment of LC3 lipidation by western blotting. ACTB was used as a loading control. (d) H4-GFP-LC3 cells were maintained in control conditions and treated with HDA alone or in combination with 100 nM BAF.A1 for 0.5, 1, 2, or 4 h. Thereafter, the number of cytoplasmic GFP-LC3^+^ dots per cell was quantified by fluorescence microscopy. Data are the means ± SD of at least three independent experiments (***p<0.001 versus untreated cells) (^#^p<, ^###^p<0.001 versus cells treated with BAF.A1. (e, f) *Wild-type* (WT) or *Atg5*^*-/-*^ MEFs (e) and Control (Co) or shATG5 SH-SY5Y cells (f) were cultured in control conditions (Co), incubated with 10 nM rapamycin (RAPA) (e), EBSS (f) or treated with 25 and 50 μM QBA or 25 and 50 μM HDA for 4 h, followed by the assessment of LC3 lipidation. ATG5 levels were monitored as a genotype control, and ACTB (e) or GAPDH (f) levels was used as a loading control. Densitometry was employed to quantify the abundance of lipidated LC3 (LC3-II). (PDF 654 kb)

Fig S2 **Implication of mTOR in QBA-induced autophagy**. (a, b) H4 cells were transfected with siUNR or with siRNA specific for TSC2 (siTSC2) for 48 h, and either maintained in control conditions (Co) or treated with 50 μM QBA or 50 μM HDA for 4 h. Thereafter, S6K phosphorylation were assessed by immunoblotting. TSC2, S6K and ACTB levels were monitored as a genotype control and to ensure equal loading of lanes (a). Densitometry was employed to quantify the abundance of p-S6K (b). (PDF 183 kb)

Fig S3 **Modulation of protein acetylation mediated by QBA is SIRT1-dependent. (**a, b) SHSY5Y cells were cultured in control conditions (Co) or incubated with EBSS medium or treated with 50 μM QBA or 50 μM HDA for 4 h. Thereafter, cells were processed for the assessment of Ac-K by western blotting (a) and immunofluorescence (b). Scale bar= 10 μm. (**c**) H4-GFP-LC3 cells were cultured in control conditions (Co) or treated with 50 μM QBA or 50 μM HDA, alone or combined with 2 μM EX527 for 4 h. Thereafter, LC3 was immunoprecipitated from cell lysates with GFP antibody and analyzed by immunoblotting using Ac-K and GFP antibodies. (PDF 240 kb)

Fig S4 **Analysis of QBA-induced autophagy in vivo**. a-c) ICR mice were injected i.p. with vehicle only, 10 mg/kg QBA (a-c), or 10 mg/kg HDA (a, b) for 4 h, or mice were deprived of food (a, b) for 24 h (starvation, ST). After treatments, animals were euthanatized, and LC3 lipidation was assessed by immunoblotting in the indicated tissues. Symbols indicate significant, *p<, ** p< 0.01 and ***p˂0.001 compared with untreated mice. (PDF 271 kb).

Fig S5 **QBA decreases 6-OHDA-mediated toxicity in N2a cells. **a, b N2a cells were cultured in control conditions (Co), or pretreated with 50 μM QBA (24 h) alone or combined with 35 μM 6-hydroxydopamine (6-OHDA) for 18 h. (a) Cleaved caspase 3 (c-Casp3), cleaved PARP (c-PARP) and PARP were assessed by immunoblotting. ACTB was used as a loading control and densitometry was employed to quantify the abundance of c-Casp3 and c-PARP (b). (c, d) N2a cells were cultured in control conditions (Co), or pretreated with 50 μM QBA for 24 h, followed by the treatment with 35 μM 6-hydroxydopamine (6-OHDA) alone or combined with 10 mM 3-methyladenine (3-MA). Eighteen hours later, the percentage of Annexin V-FITC (c) and PI-positive cells (d) was evaluated by flow cytometry (n=10000 events). Columns indicate means ± SD. Symbols indicate significant, ***P ˂ 0.001 and non significant (n.s.) compared with the respective untreated groups. # P<, ###P< 0.001 and non significant (n.s.) compared with 6-OHDA treated cells. (PDF 317 kb)

## Data Availability

The datasets generated during and/or analyzed during the current study are available from the corresponding author on reasonable request.

## Abbreviations

AMPK: AMP-activated protein kinase
ATG: Autophagy-related
BAF.A1: Bafilomycin A1
BECN1: Beclin-1
CR: Caloric restriction
FOXO1: Forkhead box protein O 1
HDA: 10-Hydroxydecanoic acid
6-OHDA: 6-Hydroxydopamine
LAMP2: Lysosomal-associated membrane protein-2
mTOR: Mammalian target of rapamycin
PD: Parkinson’s disease
PI: Propidium iodide
QBA: Queen bee acid
PI3K-III: Class III phosphatidylinositol- 3-kinase
PI3P: Phosphatidylinositol 3 phosphate
RJ: Royal Jelly
SIRT1: Sirtuin 1 or silent information regulator 2 homolog 1
S6K: S6 kinase
SQSTM1: Sequestosome 1
TSC2: Tuberous sclerosis complex 2
