# Supplementary figures and images for: Neuroprotective properties of queen bee acid by autophagy induction
Source: Cell Biol Toxicol. 2021 Aug 27;39(3):751–70. doi: 10.1007/s10565-021-09625-w (PMC10406658; doi:10.1007/s10565-021-09625-w)

**a**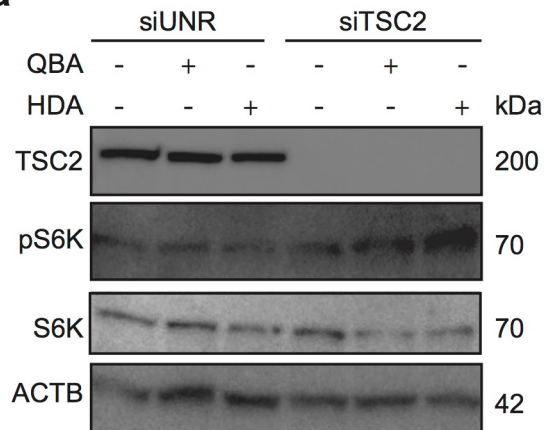**b**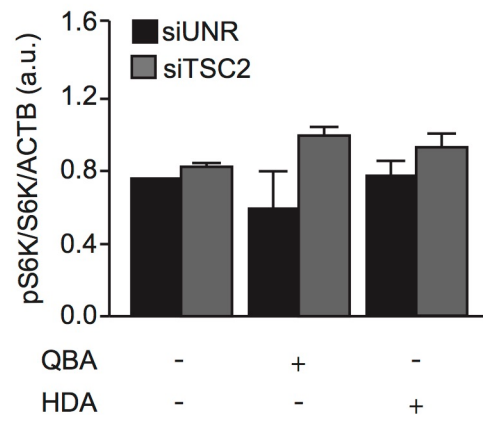

Supplement: Supplementary file 1 — Lipid fraction of RJ induces autophagy. (a) U251 cells were cultured in control conditions (Co), incubated with EBSS medium, or treated with QBA (10, 25, 50, 100, 250, 500 and 1000 μM) for 4 h followed by the assessment of LC3 lipidation by western blot. ACTB levels were used as a loading control. (b) U251 cells were cultured in control conditions (Co), incubated with 10 nM rapamycin (RAPA), or treated with QBA (250 and 500 μM) alone or in combination with 100 nM BAF.A1 for 4 h followed by the assessment of LC3 lipidation by western blot. ACTB levels were used as a loading control. (c) H4 cells were cultured in control conditions (Co), incubated with 10 nM rapamycin (RAPA), or treated with 50 μM QBA or 50 μM HDA alone or in combination with 100 nM BAF.A1 for 4h followed by the assessment of LC3 lipidation by western blotting. ACTB was used as a loading control. (d) H4-GFP-LC3 cells were maintained in control conditions and treated with HDA alone or in combination with 100 nM BAF.A1 for 0.5, 1, 2, or 4 h. Thereafter, the number of cytoplasmic GFP-LC3+ dots per cell was quantified by fluorescence microscopy. Data are the means ± SD of at least three independent experiments (***p<0.001 versus untreated cells) (#p<, ###p<0.001 versus cells treated with BAF.A1. (e, f) Wild-type (WT) or Atg5-/- MEFs (e) and Control (Co) or shATG5 SH-SY5Y cells (f) were cultured in control conditions (Co), incubated with 10 nM rapamycin (RAPA) (e), EBSS (f) or treated with 25 and 50 μM QBA or 25 and 50 μM HDA for 4 h, followed by the assessment of LC3 lipidation. ATG5 levels were monitored as a genotype control, and ACTB (e) or GAPDH (f) levels was used as a loading control. Densitometry was employed to quantify the abundance of lipidated LC3 (LC3-II). (PDF 654 kb) [file 10565_2021_9625_MOESM1_ESM.pdf]

**a**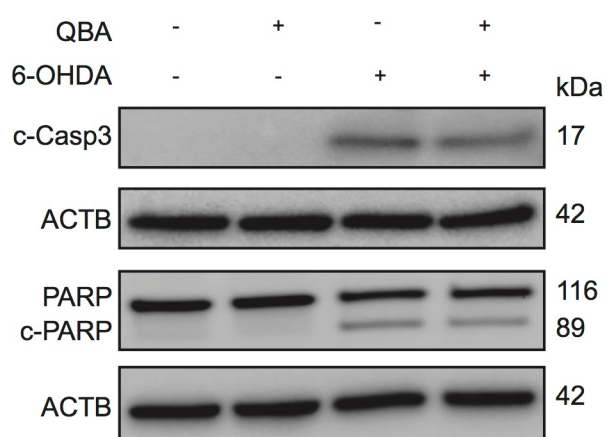**b**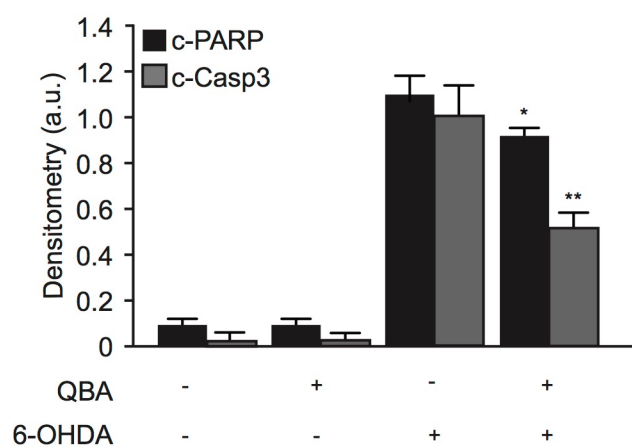**c**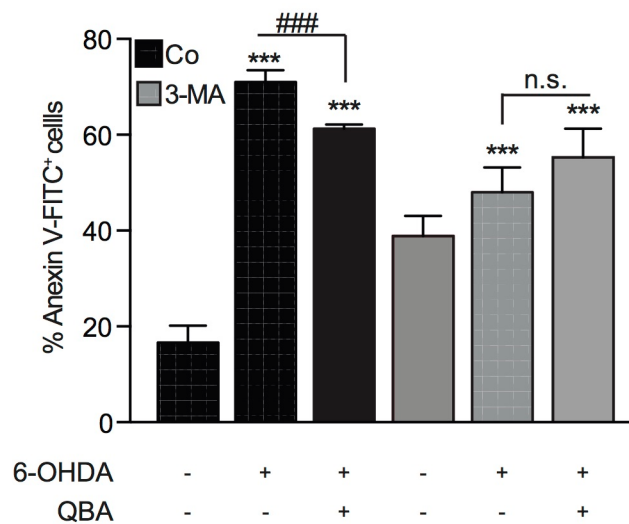**d**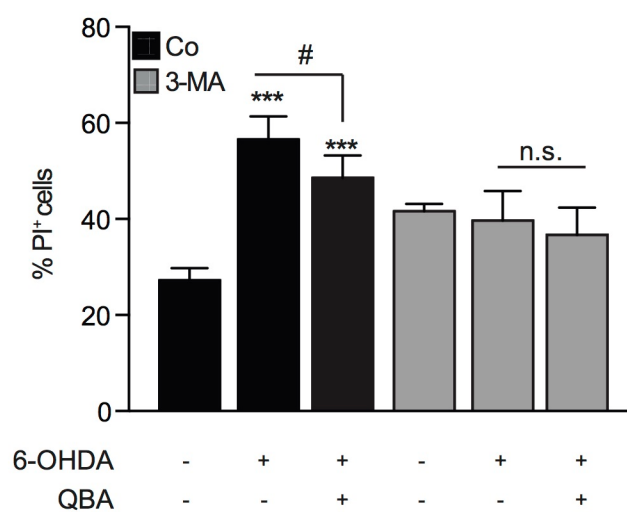

Supplement: Supplementary file 2 — Implication of mTOR in QBA-induced autophagy. (a, b) H4 cells were transfected with siUNR or with siRNA specific for TSC2 (siTSC2) for 48 h, and either maintained in control conditions (Co) or treated with 50 μM QBA or 50 μM HDA for 4 h. Thereafter, S6K phosphorylation were assessed by immunoblotting. TSC2, S6K and ACTB levels were monitored as a genotype control and to ensure equal loading of lanes (a). Densitometry was employed to quantify the abundance of p-S6K (b). (PDF 183 kb) [file 10565_2021_9625_MOESM2_ESM.pdf]

**a**

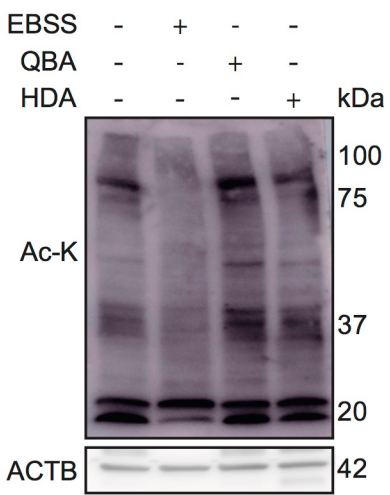

**b**

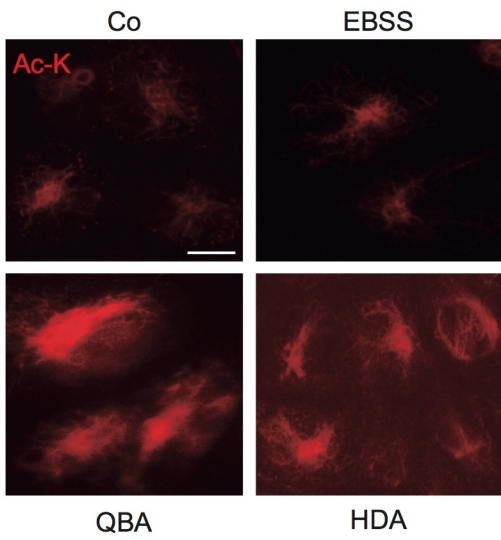

**c**

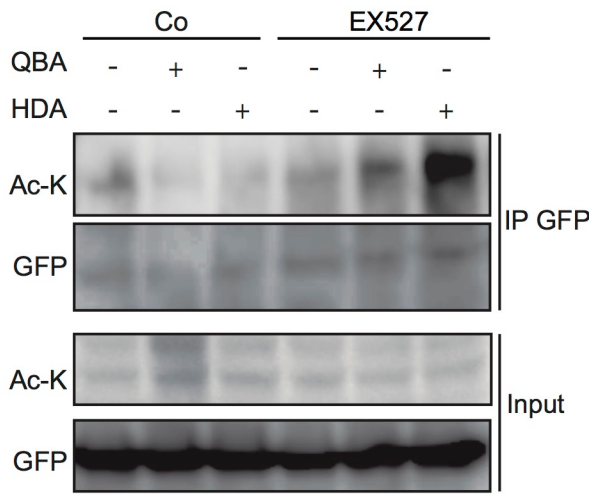

Supplement: Supplementary file 3 — Modulation of protein acetylation mediated by QBA is SIRT1-dependent. (a, b) SHSY5Y cells were cultured in control conditions (Co) or incubated with EBSS medium or treated with 50 μM QBA or 50 μM HDA for 4 h. Thereafter, cells were processed for the assessment of Ac-K by western blotting (a) and immunofluorescence (b). Scale bar= 10 μm. (c) H4-GFP-LC3 cells were cultured in control conditions (Co) or treated with 50 μM QBA or 50 μM HDA, alone or combined with 2 μM EX527 for 4 h. Thereafter, LC3 was immunoprecipitated from cell lysates with GFP antibody and analyzed by immunoblotting using Ac-K and GFP antibodies. (PDF 240 kb) [file 10565_2021_9625_MOESM3_ESM.pdf]

**a**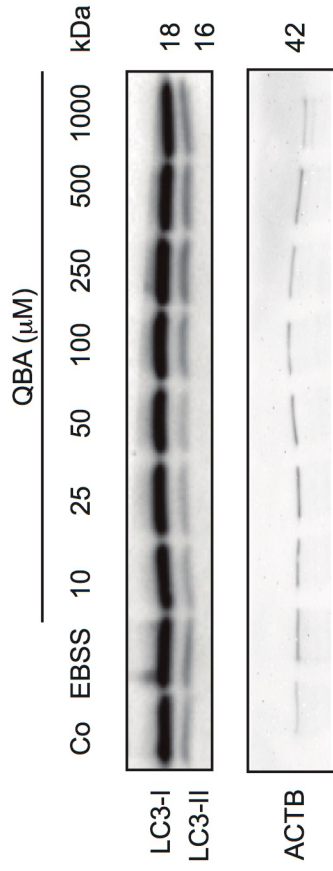**b**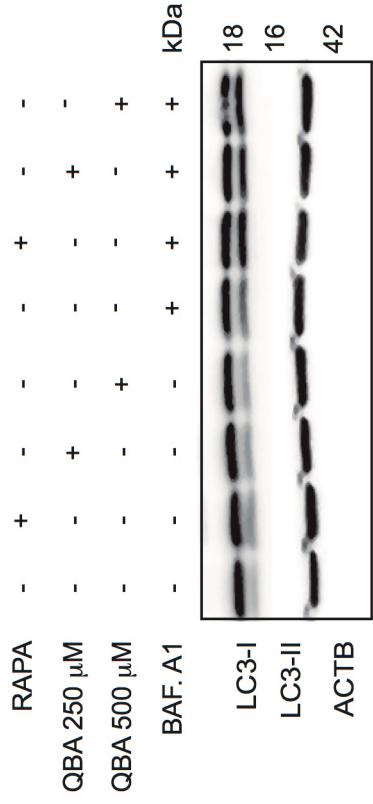**c**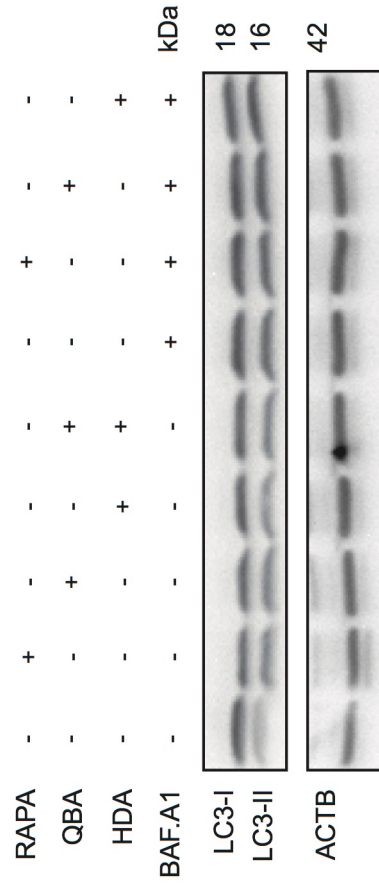**d**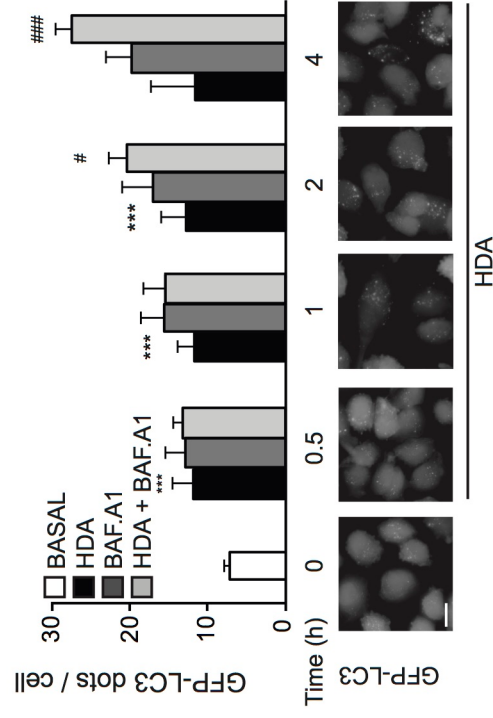**e**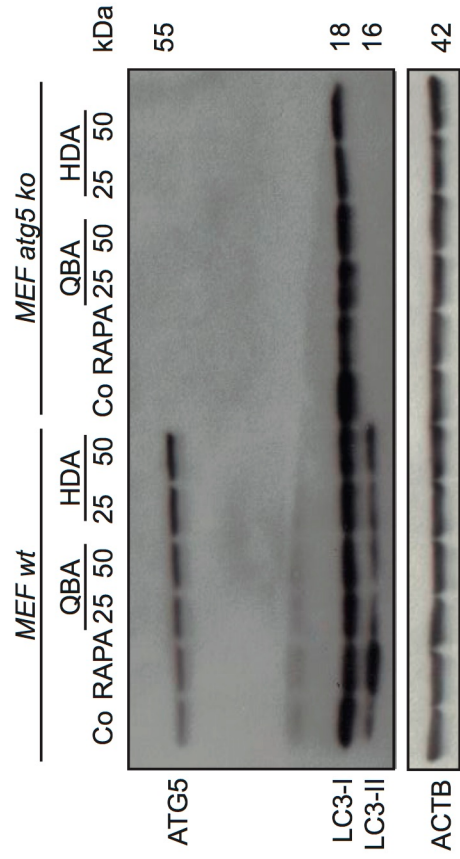**f**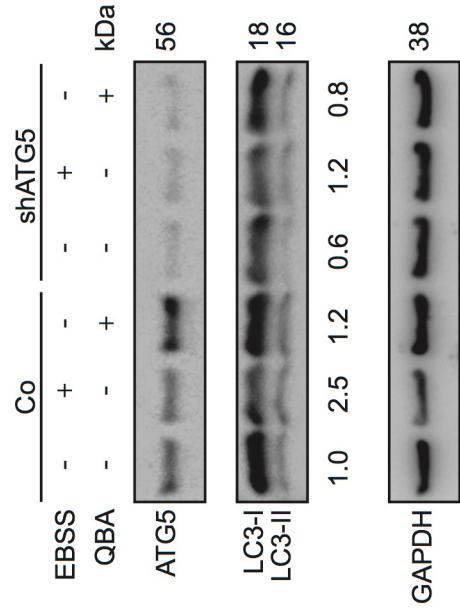

Supplement: Supplementary file 4 — Analysis of QBA-induced autophagy in vivo. a-c) ICR mice were injected i.p. with vehicle only, 10 mg/kg QBA (a-c), or 10 mg/kg HDA (a, b) for 4 h, or mice were deprived of food (a, b) for 24 h (starvation, ST). After treatments, animals were euthanatized, and LC3 lipidation was assessed by immunoblotting in the indicated tissues. Symbols indicate significant, *p<, ** p< 0.01 and ***p˂0.001 compared with untreated mice. (PDF 271 kb). [file 10565_2021_9625_MOESM4_ESM.pdf]

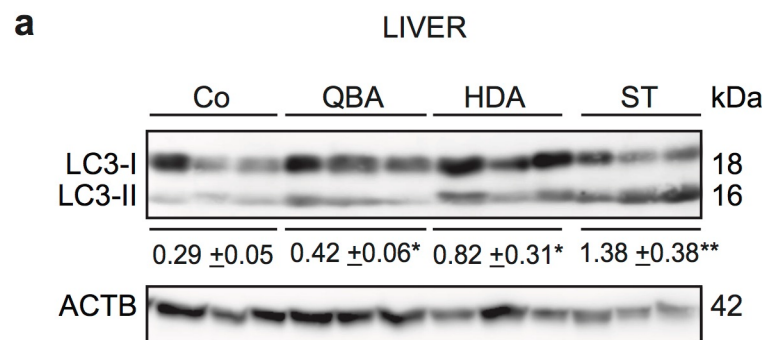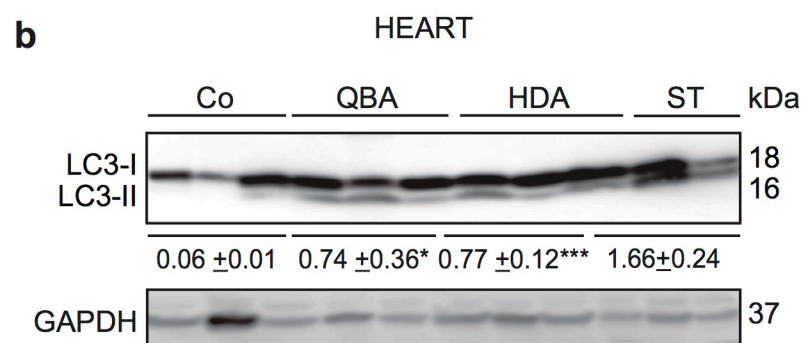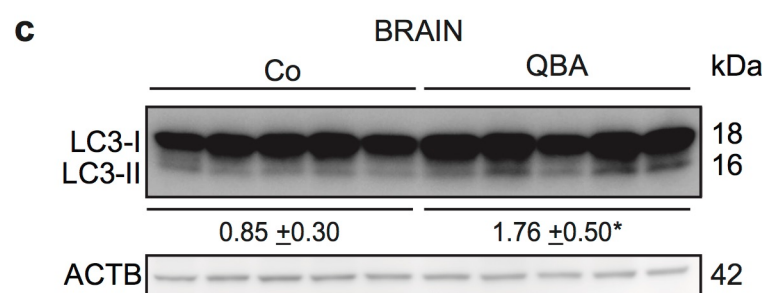

Supplement: Supplementary file 5 — QBA decreases 6-OHDA-mediated toxicity in N2a cells. a, b N2a cells were cultured in control conditions (Co), or pretreated with 50 μM QBA (24 h) alone or combined with 35 μM 6-hydroxydopamine (6-OHDA) for 18 h. (a) Cleaved caspase 3 (c-Casp3), cleaved PARP (c-PARP) and PARP were assessed by immunoblotting. ACTB was used as a loading control and densitometry was employed to quantify the abundance of c-Casp3 and c-PARP (b). (c, d) N2a cells were cultured in control conditions (Co), or pretreated with 50 μM QBA for 24 h, followed by the treatment with 35 μM 6-hydroxydopamine (6-OHDA) alone or combined with 10 mM 3-methyladenine (3-MA). Eighteen hours later, the percentage of Annexin V-FITC (c) and PI-positive cells (d) was evaluated by flow cytometry (n=10000 events). Columns indicate means ± SD. Symbols indicate significant, ***P ˂ 0.001 and non significant (n.s.) compared with the respective untreated groups. # P<, ###P< 0.001 and non significant (n.s.) compared with 6-OHDA treated cells. (PDF 317 kb) [file 10565_2021_9625_MOESM5_ESM.pdf]
